# A prospective evaluation of rivaroxaban on haemostatic parameters in apparently healthy dogs

**DOI:** 10.1002/vms3.161

**Published:** 2019-03-08

**Authors:** Liam A. Evans, Colleen Tansey, Melissa Wiebe, Caroline Q. Sloan, Jeffrey E. Patlogar, Sarah Northcutt, Lisa A. Murphy, Reid K. Nakamura

**Affiliations:** ^1^ Inland Valley Veterinary Specialists and Emergency Center Upland California USA; ^2^ Colorado State University Fort Collins Colorado USA; ^3^ Veterinary Specialty Center of Delaware New Castle Delaware USA; ^4^ Idexx laboratories Westbrook Maine USA

**Keywords:** coagulation, embolism, thromboembolism

## Abstract

The purpose of this study was to determine the effect of rivaroxaban (RIV) on haemostatic parameters assessed by prothrombin time (PT), activated partial thromboplastin time (aPTT) and kaolin‐activated thromboelastography (TEG) in apparently healthy dogs administered 1 mg kg^−1^ orally once daily for 1 week. Eleven dogs had a baseline complete blood count (CBC), fibrinogen, platelet count, serum chemistry profile, PT, aPTT, and TEG performed. Each dog was then administered approximately 1.0 mg kg^−1^ of RIV orally once daily for 1 week and the CBC, fibrinogen, platelet count, serum chemistry profile, PT, aPTT, and TEG was re‐evaluated. Any side effects attributed to RIV were noted at this time. One dog was excluded due to identification of a macrocytic thrombocytopenia on pre‐treatment blood work. The remaining 10 enrolled dogs completed the study. Dogs received a median dose of 1.02 mg kg^−1^ (range 0.94–1.17 mg kg^−1^) of RIV once daily and was associated with a significant increase in pulse, packed cell volume, total solids, platelet count, fibrinogen and a significant decrease in mean corpuscular haemoglobin and mean corpuscular haemoglobin concentration. There was no significant change in PT, aPTT or any TEG parameters. The RIV appeared well tolerated with one dog having one episode of vomiting on day 4 but otherwise no other side effects were identified clinically or on recheck blood work. The results of this study suggests that RIV at a dose of 1 mg kg^−1^ orally once daily is safe and well tolerated but does not cause a significant prolongation of PT, aPTT or TEG parameters.

## Introduction

Rivaroxaban (RIV) is an orally administered factor Xa inhibitor that has been shown to be effective and safe for preventing venous thromboembolism in people with a reduced risk of bleeding complications compared to warfarin (Weinz *et al*. 2009; Patel *et al*. 2011; Samama 2011; Turpie *et al*. 2011; Mega *et al*. 2012,). RIV has predictable pharmacokinetics and pharmacodynamics and thus routine laboratory monitoring is not required in people (Weinz *et al*. 2009; Mavrakanas & Bounameaux 2011; Flierl *et al*. 2013). Furthermore, RIV is administered orally once daily thereby bypassing the need for multiple daily injections as is required with heparin administration (Mavrakanas & Bounameaux 2011; Turpie *et al*. 2011).

RIV was found to have a peak anticoagulant effect 1–2 h after oral administration in healthy dogs and is associated with a significant increase in PT and thrombin generation parameters at a dose of 2 mg kg^−1^ orally once or twice daily (Conversy *et al*. 2017). The magnitude of effect on haemostatic parameters was not significantly different between once or twice daily dosing although the twice daily dosing regimen was found to produce a more prolonged anticoagulant effect (Conversy *et al*. 2017). Interestingly, RIV use in dogs was not associated with a significant prolongation of TEG parameters (Conversy *et al*. 2017) which is contrary to humans receiving therapeutic levels of RIV where increased R and K values and significantly decreased G, maximum amplitude, alpha angle and LY30 compared to baseline have been reported (Bowry *et al*. 2014).

There have been two clinical reports evaluating RIV in dogs with thromboembolic disorders and both found that RIV appeared safe and well tolerated but it was unclear if RIV produced a clinical anticoagulant effect (Morassi *et al*. 2016; Yang *et al*. 2016). In both reports, the maximum dosage of RIV was approximately 1 mg kg^−1^ orally once daily or half the dose of what had been evaluated previously (Conversy *et al*. 2017). Consequently, the minimum dose of RIV required to produce a measurable anticoagulant effect in dogs is unclear. As such, the purpose of this study was to determine if RIV administered at 1 mg kg^−1^ orally once daily in a group of apparently healthy dogs produced a significant change in PT, aPTT or TEG parameters. The secondary end point was to evaluate if RIV use was associated with any clinical or laboratory side effects as measured by clinical signs and recheck blood work after 1 week of use. The hypothesis of this study was that RIV at a dose of 1 mg kg^−1^ orally once daily would produce a significant prolongation in PT without affecting TEG parameters with no identifiable clinical or biochemical side effects.

## Materials and methods

The study protocol was approved by the Institutional Animal Care and Use Committee. Informed client consent was obtained in all cases. Eleven dogs were enrolled. This number was selected given a previous veterinary study found a significant change in PT in 8 dogs receiving RIV at 2 mg kg^−1^ orally once or twice daily (Conversy *et al*. 2017) and another study found a significant change in TEG parameters in 10 people receiving RIV compared to their baseline values (Bowry *et al*. 2014). Power calculation to determine sample size was not performed. Dogs were deemed healthy based on an absence of clinical signs or previous medical conditions, and normal physical examination and baseline complete blood count (CBC) (Idexx Procyte Hematology Analyzer, Sysmex Corporation) and serum chemistry profile and fibrinogen (Idexx Catalyst One Analyzer, Idexx Laboratories, Inc). Dog were eligible if they were greater than 12 months of age at the time of study initiation were apparently healthy. Dogs were ineligible if they were receiving medications other than heartworm preventative or flea and tick preventative. The dogs were restrained and blood samples obtained from the jugular vein in all study participants. The blood was collected into plastic blood tubes with 3.2% buffered sodium citrate solution in a 1:9 ratio following collection of a discard volume as per current recommendations (Flatland *et al*. 2014). All blood samples were stored at room temperature for thirty minutes prior to the TEG (Thrombelastograph Analyser 5000, Haemoscope Corp., Niles, IL at 37C) being performed by the principal investigator. A baseline PT and aPTT (VetScan VS Pro PT and aPTT Combination test, Abaxis) was also performed at this time. Owners were then instructed to administer RIV (Rivaroxaban, Xarelto, Bayer HealthCare AG, Leverkusen, Germany) at an approximate dose of 1 mg kg^−1^ orally once daily with food. At the end of the study (7 days) period, the owners were instructed to return with their dog and the remaining RIV tablets. At this time, the remaining tablets were counted to verify compliance to the study protocol. The CBC, serum chemistry profile, fibrinogen, PT and aPTT and TEG was again repeated at this time by the same principal investigator in an identical manner described previously. The blood was collected approximately 1–2 h after last oral RIV dose administration to coincide with maximal RIV effect on haemostatic parameters (Conversy *et al*. 2017). Data collected included age, sex, breed, bodyweight (kg), temperature, heart rate, respiratory rate and side effects possibly attributed to RIV administration and TEG parameters before and after RIV administration.

In accordance with the current TEG recommendations (deLaforcade *et al*. 2014; Flatland *et al*. 2014), 1 mL of citrated whole blood was placed in a 1% kaolin vial, which was then inverted five times to ensure appropriate sample activation. After activation, 340 μL of citrated whole blood and 20 μL of CaCl_2_ were pipetted into a cuvette used in the TEG analyzer.^c^ The TEG tracing was automatically stopped at 60 min after the MA was recorded. The TEG results were generated by analyzer's software and included 10 variables (R, K, alpha, MA, G, CI, LY30, A30, LY60, and A60) (Hanel *et al*. 2014). Quality assurance testing was completed every 8 h of RIV testing. An owner questionnaire was provided at the end of the study (Day 7) for each patient enrolled with regard to adverse events, including lethargy, vomiting, diarrhoea, lameness, change in appetite or behavioural changes.

### Statistical analysis

All data were evaluated for normality using a D'Agostino and Pearson normality test, Shapiro–Wilk, and Kolmogorov–Smirnov normality test. If any data failed one of these tests then data were treated as non‐parametric. A paired *t‐*test was used for data that was normally distributed and a Wilcoxon matched pairs signed‐rank test for data that was not normally distributed. No post hoc analysis was performed. For this analysis a *P* value < 0.05 was considered statistically significant. Statistical analyses were performed using a statistical software package (Sergeant, ESG, 2017. Epitools epidemiological calculators. Ausvet Pty Ltd. Bruce ACT 2617, Australia).

## Results

Eleven dogs were prospectively enrolled in the study. One dog was excluded from the study after identification of a macrocytic thrombocytopenia on pre‐treatment CBC. The remaining ten dogs were enrolled and completed the study. Breeds included in the study were Labrador Retriever (*n* = 2), Staffordshire Terrier (*n* = 2), German Shorthaired Pointer (*n* = 1), Labrador Mix (*n* = 1), Australian Shepherd (*n* = 1), Husky Mix (*n* = 1), Queensland Healer (*n* = 1), and Boston Terrier (*n* = 1) There were seven castrated males, two spayed females and one intact male. Median age was 48 months (range 24–60 months). The median dose of RIV in this study was 1.02 mg kg^−1^ (range 0.94–1.17 mg kg^−1^). Pre‐ and post‐treatment physical examination, CBC and serum chemistry profile parameters are shown in Tables 1, 2, 3. Most dogs were panting, both pre‐ and post‐treatment which prevented statistical analysis of the respiratory rate. There was a significant increase in pulse rate, mean corpuscular haemoglobin (MCH), mean corpuscular haemoglobin concentration (MCHC), total protein, and platelet count after RIV administration. While these changes were statistically significant, they did not appear to result in any significant clinical effect on the study participants. There were no other significant changes in any other physical examination or haematologic parameters.

**Table 1:** Physical examination parameters pre‐ and post‐treatment in 10 dogs receiving 1 mg kg^−1^ orally of Rivaroxaban once daily for 1 week

Parameter (units)	Pre‐treatment	Post‐treatment	*P* value
Weight (kg)	23.74 (6.69)	23.88 (6.85)	0.27
Temperature (C)	38.8 (0.48)	38.8 (0.53)	0.59
Pulse (beats per minute)	111.6 (23.36)	131.4 (24.13)	**0.04**
Systolic blood pressure (mm Hg)	112.3 (28.28)	100.7 (32)	0.43

If data were analysed with a paired *t*‐est, we reported a mean and standard deviation. If data were analysed with a Wilcoxon matched pairs signed‐rank test, the median and interquartile range (25th and 75th percentile) is reported. Bold indicates statistically significant *P* value.

**Table 2:** Complete blood count parameters pre‐ and post‐treatment in 10 dogs receiving 1 mg kg^−1^ orally of Rivaroxaban once daily for 1 week

Parameter (units)	Pre‐treatment	Post‐treatment	Reference range	*P* Value
Red blood cell count (x 10^12^ L^−1^)	7.65 (0.83)	7.73 (0.69)	5.65–8.87 10^12^/L	0.66
Haematocrit (L L^−1^)	0.526 (0.0615)	0.531 (0.0439)	0.373–0.617 L L^−1^	0.73
Packed cell volume (%)	51.6 (4.40)	54.6 (3.84)	(37–55%)	**0.02**
Total protein (g L^−1^)	63 (61.8–70)	72 (64–76.3)	54–85	**0.0039**
Haemoglobin (g L^−1^)	182.6 (19.1)	179.9 (13.3)	131–205	0.56
MCV (fL)	68.72 (2.56)	68.77 (2.59)	61.6–73.5	0.88
MCH (pg)	23.85 (23.6–24.55)	23.5 (22.83–24.0)	21.2–25.9	**0.0039**
MCHC (1)	34.76 (0.81)	33.9 (0.56)	32.0–37.9	**0.0039**
White blood cell count (x 10^9^ L^−1^)	9.31 (2.05)	8.55 (1.56)	5.05–16.76 x10^9^ L^−1^	0.21
Neutrophil count (x 10^9^ L^−1^)	5.29 (4.2–7.41)	4.77 (4.17–6.45)	2.95–11.64 x10^9^ L^−1^	0.37
Lymphocyte count (x 10^9^ L^−1^)	2.33 (1.53 – 2.97)	2.16 (1.57 – 2.5)	1.05–5.10	0.49
Monocyte count (x10^9^ L^−1^)	0.56 (0.47 – 0.64)	0.53 (0.42 – 0.78)	0.16–1.12	0.32
Eosinophil count (x10^9^ L^−1^)	0.52 (0.23)	0.57 (0.22)	0.06–1.23	0.16
Basophil count (x 10^9^ L^−1^)	0.03 (0.03)	0.01 (0.009)	0.00–0.10	0.1162
Platelet count (x 10^9^ L^−1^)	225 (46.09)	245 (52.91)	148–484	**0.035**

If data were analysed with a paired *t*‐test, we reported a mean and standard deviation. If data were analysed with a Wilcoxon matched pairs signed‐rank test, the median and interquartile range (25th and 75th percentile) is reported. Bold indicates statistically significant *P* values.

**Table 3:** Serum chemistry parameters pre‐ and post‐treatment in 10 dogs receiving 1 mg kg^−1^ orally of Rivaroxaban once daily for 1 week

Parameters	Pre‐treatment	Post‐treatment	Reference range	*P* value
Glucose (mmol L^−1^)	5.80 (0.56–6.24)	5.5 (5.25–5.86)	4.11–7.94	0.10
Creatinine (*μ*mol L^−1^)	122.89 (15.9)	121.99 (19.45)	44–159	0.82
Blood Urea Nitrogen (mmol L^−1^)	6.25 (5.62‐7.85)	6.06 (5.62–7.50)	2.50–8.93	0.42
Phosphorus (mmol L^−1^)	1.12 (0.32)	1.17 (0.37)	0.81–2.19	0.74
Calcium (mmol L^−1^)	2.53 (0.11)	2.51 (0.88)	1.98–3.00	0.55
Albumin (g L^−1^)	33.8 (1.8)	33.2 (3.1)	23–40	0.44
Globulin (g L^−1^)	38.5 (2.2)	37.0 (3.5)	25–45	0.14
ALT (U L^−1^)	72.4 (23.61)	66.5 (21.65)	10–125	0.15
ALKP (U L^−1^)	68.7 (45.92)	51.8 (22.84)	23–212	0.07
GGT (U L^−1^)	1 (0‐3.5)	2 (0‐7)	0–11	0.21
Total bilirubin (*μ*mol L^−1^)	5.99 (4.29)	6.16 (4.28)	0–15	0.95
Cholesterol (mmol L^−1^)	5.08 (1.16)	5.12 (1.08)	2.84–8.27	0.75
Amylase (U L^−1^)	653.5 (518–860.5)	666.5 (554.8–796.3)	500–1500	0.76
Lipase (U L^−1^)	869 (762.3–1517)	942 (760.3–1376)	200–1800	0.84

If data were analysed with a paired *t*‐test, we reported a mean and standard deviation. If data were analysed with a Wilcoxon matched pairs signed‐rank test, the median and interquartile range (25th and 75th percentile) is reported.

PT, aPTT, fibrinogen, and TEG parameters pre‐ and post‐RIV treatment are shown in Tables 4 and 5. There was a significant increase in fibrinogen after 1 week of RIV at 1 mg kg^−1^ orally once daily, however, there were no other significant change in the PT, aPTT or TEG parameters. One dog had one episode of vomiting on day 4 approximately 1 h after RIV administration but recovered without medication or supportive care; no other side effects were reported during the study period.

**Table 4:** Standard haemostatic parameters pre‐ and post‐treatment in 10 dogs receiving 1 mg kg^−1^ orally of Rivaroxaban once daily for 1 week

Parameters	Pre‐treatment	Post‐treatment	Reference range	*P* value
Fibrinogen (*μ*mol L^−1^)	1.88 (1.76–2.45)	5.12 (4.72–5.73)	1.47–6.62	**0.002**
Prothrombin time (s)	17.47 (0.94)	18.14 (1.69)	15–19 s	0.19
Activated partial thromboplastin time (s)	91.45 (86.53–98.68)	99.65 (89.25–111.8)	90–105 s	0.12

If data were analysed with a paired *t*‐test, we reported a mean and standard deviation. If data were analysed with a Wilcoxon matched‐pairs signed rank test, the median and interquartile range (25th and 75th percentile) is reported. Bold indicates statistically significant *P* value.

**Table 5:** Thromboelastographic parameters pre‐ and post‐treatment in 10 dogs receiving 1 mg kg^−1^ orally of Rivaroxaban once daily for 1 week

Thromboelastography parameter (units)	Pre‐treatment	Post‐treatment	P value
R (min)	2.65 (2.1–3.1)	3 (2.27–3.75)	0.76
K (min)	1.68 (0.60)	1.74 (0.45)	0.78
Angle (degrees)	67.08 (5.49)	67.37 (4.79)	0.86
MA (mm)	53.88 (8.48)	54.8 (7.21)	0.73
G (degrees s^−1^)	6.22 (2.33)	6.32 (1.78)	0.87
A (mm)	28.7 (11.3–45)	47.95 (36.55–51.7)	0.10
CI	1.44 (2.08)	1.29 (1.14)	0.76
LY30 (%)	10.35 (2.7–29)	0.35 (0–14.2)	0.08
A30 (mm)	44.25 (21.33)	50.68 (8.78)	0.34
LY 60 (%)	25.45 (10.73–46.90)	82.4 (1.32–20.45)	0.08
A60 (mm)	31.03 (21.79)	45.1 (9.20)	0.079

If data were analysed with a paired *t*‐test, we reported a mean and standard deviation. If data were analysed with a Wilcoxon matched pairs signed‐rank test, the median and interquartile range (25th and 75th percentile) is reported.

## Discussion

The primary finding of this study is that RIV administered at 1 mg kg^−1^ orally once daily for 1 week did not produce any significant change in PT, aPTT or TEG parameters in 10 apparently healthy dogs. As such although RIV appears to be well tolerated in accordance with the previous clinical reports (Morassi *et al*. 2016; Yang *et al*. 2016). As PT/PTT are not recommended for assessing the effectiveness of RIV and there are other more accurate tests (anti‐Xa assays) which were not performed in this study and is not widely available, it is difficult to determine if there truly was no clinical anti‐coagulant effect at this dose. Nevertheless, this is an important finding because at this time, RIV remains expensive and thus the requirement for the higher dose may potentially limit its usefulness in veterinary medicine due to cost concerns.

A previous study in people found that the measurable haemostatic effect of RIV was only produced during the peak RIV plasma levels and PT, aPTT and activated clotting time frequently were normal during trough RIV levels (Francart *et al*. 2014). It is important to note that the sensitivity of PT and aPTT for RIV is affected by the activator used. Previous studies have shown that PT is prolonged with RIV but the effect varies based on the thromboplastin reagent used (Smith & Morrissey 2007; Samama *et al*. 2008, 2010). As the thromboplastin reagent used in this study was different from the previous study, this may explain part of the absence of prolongation of PT despite apparent appropriate dosing of RIV (Conversy *et al*. 2017). The same standard activator was used at the authors’ institution to perform the PT and aPTT tests, however, it is unknown if, *in vivo* in the dog, standard coagulation assay activators are insensitive to RIV. The authors attempted to maximize the anticoagulant effect by measuring haemostatic function 1–2 h after the last dose of RIV to coincide with the peak effect of RIV (Conversy *et al*. 2017). However, the authors are unable to rule out the possibility that peak RIV levels were missed due to individual variations in metabolism and pharmacokinetics and thus may have contributed to the non‐significant change in haemostatic parameters in this study. It would have been more ideal to measure RIV levels to identify the peak plasma levels and measure clotting function at this time but measurement of RIV levels was not available at the author's institution during the study period.

In the short term, RIV appeared well tolerated and safe in agreement with previous studies (Morassi *et al*. 2016; Yang *et al*. 2016; Conversy *et al*. 2017) as there was one episode of vomiting but no biochemical abnormalities identified on CBC or serum chemistry profile. Future studies are required to ascertain if RIV remains well tolerated when administered chronically over weeks to months. Previous reports in human medicine have identified hepatotoxicity in some individuals receiving RIV although the exact frequency remains unknown (Caldeira *et al*. 2014; Cordeanu *et al*. 2016). There was no significant change in any of the hepatic enzymes on repeat serum chemistry profile to suggest a hepatotoxic reaction that occurs in dogs at the dose reported here.

There was a significant increase in fibrinogen levels in dogs receiving RIV approximately 1 mg kg^−1^ orally once daily. This was a surprising finding because previous studies in people found that RIV usually has no effect or actually lowers the fibrinogen levels by approximately 10% depending on measurement methodology (Hillarp *et al*. 2011). Interestingly, there was no change in MA which is an indicator of clot strength of which fibrinogen contributes. However, the platelet count is the strongest contributor to MA (Lang & von 2006). Furthermore, there can be some lack of correlation between TEG and other more traditional coagulation tests, including the measurement of fibrinogen, as the latter are plasma‐based assays. This is in contrast to TEG which is a whole blood assay. Fibrinogen is an acute phase protein with elevations most commonly associated with inflammation (Murata *et al*. 2004). Furthermore, there was also a significant increase in platelet count in dogs receiving RIV compared to baseline as well. However, future studies, including blood smears and flow cytometry, should be performed to assess this platelet increase and if there is any change to platelet function following RIV administration before drawing any specific conclusions regarding RIV's effect on platelets. Increases in platelet count are frequently associated with inflammation as well (Schafer 2001; Jagadesham *et al*. 2014; Matowicka‐Karna 2016; Woolcock *et al*. 2017). Previous studies in people have reported increases in platelet count and fibrinogen levels as a marker of inflammation and associated with more severe disease in various conditions including neoplasia, acute coronary syndrome, and inflammatory bowel disease (Odeberg *et al*. 2016; Suzuki *et al*. 2016). As such these findings may suggest that RIV could potentially be pro‐inflammatory in dogs. This contrasts the results from studies in people and laboratory animals where RIV has been shown to reduce inflammation via decreased thrombin generation and subsequent reduction in proteinase‐activated receptor stimulation (Dittmeier *et al*. 2016; Ellinghaus *et al*. 2016; Terry *et al*. 2016). The possibility of RIV causing inflammation is a potentially important finding as RIV is typically used in conditions where inflammation is concurrently present such as immune‐mediated haemolytic anaemia (Morassi *et al*. 2016) and therefore theoretically could worsen the underlying disease process. Although there was no significant change noted in other markers of inflammation measured in this cohort, most notably the white blood cell count and albumin levels, further evaluation into the inflammatory effects of RIV in dogs appears warranted.

Several limitations in this study here require mentioning. The sample size was small and may have contributed to a type II statistical error. It is unclear if a larger population of dogs would have identified a significant change in PT, aPTT or TEG parameters associated with RIV administration. The lack of control group may also have impeded identification of a statistically significant change in haemostatic parameters associated with RIV use as well. A previous *in vitro* study found on canine plasma that anti‐factor Xa activity and thrombin generation parameters are the most sensitive parameters for detection of RIV effects (Conversy *et al*. 2013) unfortunately such testing is not available at the authors’ institution and samples were not submitted to an outside laboratory. While the serum chemistry profiles were normal, more extensive testing (i.e. abdominal ultrasound and hepatic biopsy) was not performed to detect subclinical side effects.

In conclusion, this study indicates that RIV at a dose of 1 mg kg^−1^ orally once daily does not produce a significant change in PT, using standard activtors, aPTT or kaolin‐activated TEG parameters. There were no clinical or haematologic side effects associated with RIV administration in any of the dogs. It appears that RIV needs to be dosed higher than 1 mg kg^−1^ orally once daily to produce a measurable anticoagulant effect in dogs. The effects of RIV on inflammatory biomarkers in dogs may require further investigation.

## Source of funding

There was no external funding for this report.

## Conflict of interest

The authors report no conflicts of interest.

## Ethics statement

The authors confirm that the ethical policies of the journal, as noted on the journal's author guidelines page, have been adhered to and the appropriate ethical review committee approval has been received. The US National Research Council's guidelines for the Care and Use of Laboratory Animals were followed.

## Contributions

RN conceived the study. LE, LM, CS and RN wrote the manuscript. LE, CT, MW, JP, and SN performed the experiments and data collections. All authors reviewed, revised and accepted the manuscript.
